# Early experience with robotic pancreatoduodenectomy *versus* open pancreatoduodenectomy: nationwide propensity-score-matched analysis

**DOI:** 10.1093/bjs/znae043

**Published:** 2024-02-27

**Authors:** Nine de Graaf, Maurice J W Zwart, Jony van Hilst, Bram van den Broek, Bert A Bonsing, Olivier R Busch, Peter-Paul L O Coene, Freek Daams, Susan van Dieren, Casper H J van Eijck, Sebastiaan Festen, Ignace H J T de Hingh, Daan J Lips, Misha D P Luyer, J Sven D Mieog, Hjalmar C van Santvoort, George P van der Schelling, Martijn W J Stommel, Roeland F de Wilde, I Quintus Molenaar, Bas Groot Koerkamp, Marc G Besselink

**Affiliations:** Department of Surgery, Amsterdam UMC, location University of Amsterdam, Amsterdam, The Netherlands; Cancer Centre Amsterdam, Amsterdam, The Netherlands; Department of General Surgery, Fondazione Poliambulanza Istituto Ospedaliero, Brescia, Italy; Department of Surgery, Amsterdam UMC, location University of Amsterdam, Amsterdam, The Netherlands; Cancer Centre Amsterdam, Amsterdam, The Netherlands; Department of Surgery, Amsterdam UMC, location University of Amsterdam, Amsterdam, The Netherlands; Cancer Centre Amsterdam, Amsterdam, The Netherlands; Department of Surgery, OLVG, Amsterdam, The Netherlands; Department of Surgery, Erasmus MC Cancer Institute, Rotterdam, The Netherlands; Department of Surgery, Leiden University Medical Centre, Leiden, The Netherlands; Department of Surgery, Amsterdam UMC, location University of Amsterdam, Amsterdam, The Netherlands; Cancer Centre Amsterdam, Amsterdam, The Netherlands; Department of Surgery, Maasstad Ziekenhuis, Rotterdam, The Netherlands; Department of Surgery, Amsterdam UMC, location University of Amsterdam, Amsterdam, The Netherlands; Cancer Centre Amsterdam, Amsterdam, The Netherlands; Department of Surgery, Amsterdam UMC, location University of Amsterdam, Amsterdam, The Netherlands; Cancer Centre Amsterdam, Amsterdam, The Netherlands; Epidemiologist Department of Surgery, Amsterdam UMC, location AMC, Amsterdam, The Netherlands; Department of Surgery, Erasmus MC Cancer Institute, Rotterdam, The Netherlands; Department of Surgery, OLVG, Amsterdam, The Netherlands; Department of Surgery, Catharina Hospital, Eindhoven, The Netherlands; Department of Surgery, Medisch Spectrum Twente, Enschede, The Netherlands; Department of Surgery, Catharina Hospital, Eindhoven, The Netherlands; Department of Surgery, Leiden University Medical Centre, Leiden, The Netherlands; Department of Surgery, St Antonius Hospital, Nieuwegein, The Netherlands; Department of Surgery, Regional Academic Cancer Centre Utrecht, University Medical Centre Utrecht, Utrecht, The Netherlands; Department of Surgery, Amphia Ziekenhuis, Breda, The Netherlands; Deptartment of Surgery, Radboud University Medical Centre, Nijmegen, The Netherlands; Department of Surgery, Erasmus MC Cancer Institute, Rotterdam, The Netherlands; Department of Surgery, St Antonius Hospital, Nieuwegein, The Netherlands; Department of Surgery, Regional Academic Cancer Centre Utrecht, University Medical Centre Utrecht, Utrecht, The Netherlands; Department of Surgery, Erasmus MC Cancer Institute, Rotterdam, The Netherlands; Department of Surgery, Amsterdam UMC, location University of Amsterdam, Amsterdam, The Netherlands; Cancer Centre Amsterdam, Amsterdam, The Netherlands

## Abstract

**Background:**

Although robotic pancreatoduodenectomy has shown promising outcomes in experienced high-volume centres, it is unclear whether implementation on a nationwide scale is safe and beneficial. The aim of this study was to compare the outcomes of the early experience with robotic pancreatoduodenectomy *versus* open pancreatoduodenectomy in the Netherlands.

**Methods:**

This was a nationwide retrospective cohort study of all consecutive patients who underwent robotic pancreatoduodenectomy or open pancreatoduodenectomy who were registered in the mandatory Dutch Pancreatic Cancer Audit (18 centres, 2014–2021), starting from the first robotic pancreatoduodenectomy procedure per centre. The main endpoints were major complications (Clavien–Dindo grade greater than or equal to III) and in-hospital/30-day mortality. Propensity-score matching (1 : 1) was used to minimize selection bias.

**Results:**

Overall, 701 patients who underwent robotic pancreatoduodenectomy and 4447 patients who underwent open pancreatoduodenectomy were included. Among the eight centres that performed robotic pancreatoduodenectomy, the median robotic pancreatoduodenectomy experience was 86 (range 48–149), with a 7.3% conversion rate. After matching (698 robotic pancreatoduodenectomy patients *versus* 698 open pancreatoduodenectomy control patients), no significant differences were found in major complications (40.3% *versus* 36.2% respectively; *P* = 0.186), in-hospital/30-day mortality (4.0% *versus* 3.1% respectively; *P* = 0.326), and postoperative pancreatic fistula grade B/C (24.9% *versus* 23.5% respectively; *P* = 0.578). Robotic pancreatoduodenectomy was associated with a longer operating time (359 min *versus* 301 min; *P* < 0.001), less intraoperative blood loss (200 ml *versus* 500 ml; *P* < 0.001), fewer wound infections (7.4% *versus* 12.2%; *P* = 0.008), and a shorter hospital stay (11 days *versus* 12 days; *P* < 0.001). Centres performing greater than or equal to 20 robotic pancreatoduodenectomies annually had a lower mortality rate (2.9% *versus* 7.3%; *P* = 0.009) and a lower conversion rate (6.3% *versus* 11.2%; *P* = 0.032).

**Conclusion:**

This study indicates that robotic pancreatoduodenectomy was safely implemented nationwide, without significant differences in major morbidity and mortality compared with matched open pancreatoduodenectomy patients. Randomized trials should be carried out to verify these findings and confirm the observed benefits of robotic pancreatoduodenectomy *versus* open pancreatoduodenectomy.

## Teaser

This nationwide study in 18 centres compared the surgical outcomes of 698 patients who underwent robotic pancreatoduodenectomy with those of 698 propensity-score-matched patients who underwent open pancreatoduodenectomy. The rates of major complications and mortality did not differ significantly, whereas robotic pancreatoduodenectomy was associated with less intraoperative blood loss, a longer operating time, a shorter hospital stay, fewer grade B/C chyle leaks, and fewer wound infections than open pancreatoduodenectomy.

## Introduction

Pancreatoduodenectomy (PD) is a complex procedure associated with a high risk of postoperative complications. Robotic pancreatoduodenectomy (RPD) has gained popularity based on reports from a few experienced, very high-volume centres^1–3^. RPD aims to reduce surgical trauma compared with open pancreatoduodenectomy (OPD) and hence could improve short- and long-term outcomes. However, some studies have reported safety concerns regarding the implementation of RPD into clinical practice^4–6^.

To facilitate the safe implementation of RPD in the Netherlands, the nationwide LAELAPS-3 training programme was performed in close collaboration with the University of Pittsburgh Medical Center (UPMC) group. This programme included virtual reality and artificial organ training, followed by on-site proctoring during the first RPD procedures^7^. Although early results from the programme seemed promising in selected patients, a direct comparison with OPD is lacking^8,9^. Randomized controlled trials comparing RPD and OPD are currently lacking and the existing comparative studies are often small retrospective single-centre studies, prone to treatment allocation bias^10–12^. This bias can go both ways; outcomes of RPD can appear better, because of the selection of fit patients early in the learning curve, but also worse, because of the selection of patients with small tumours (for example neuroendocrine tumours with a soft pancreas and/or small duct) and the inclusion of the learning curve effect.

Population-based propensity-score-matched studies comparing outcomes of RPD and OPD from the start of implementation into clinical practice have not been performed. Therefore, it is unclear whether the promising results of RPD from high-volume centres can be reproduced on a nationwide scale. Comparing the outcomes of RPD and OPD is needed to confirm the safety of implementing RPD on a large scale, especially during the learning curve^13^. The aim of this study was to assess the nationwide short-term surgical outcomes of RPD in the Netherlands, from implementation in eight centres during the past 6 years to current practice, and to compare these outcomes with those of OPD using a propensity-score-matched study design.

## Methods

**Fig. 1:**
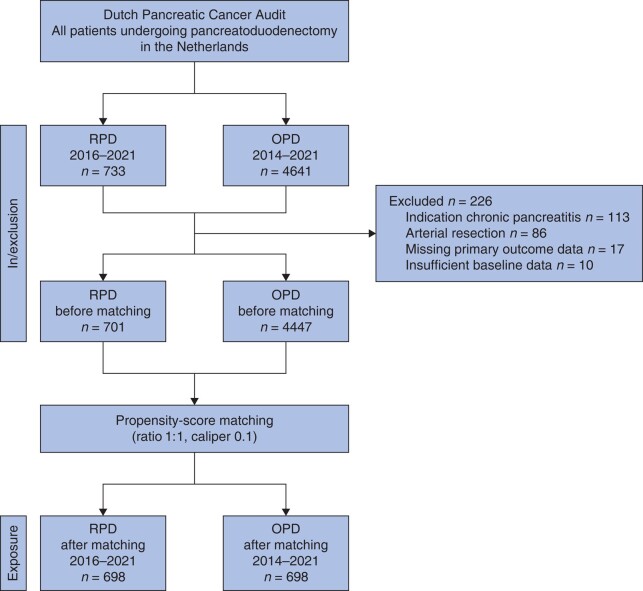
Study flow chart of included patients RPD, robotic pancreatoduodenectomy; OPD, open pancreatoduodenectomy.

A multicentre propensity-score-matched retrospective cohort study was performed using data from the prospective and mandatory Dutch Pancreatic Cancer Audit (DPCA)^14^. All data were collected by trained medical staff. The DPCA database has been verified and the completeness of the data is greater than 90% (case ascertainment) and the accuracy of the data is greater than 95%^14^. For each patient, the DPCA collects the originally planned approach (open, laparoscopic, or robotic), as well as whether the surgery was converted to an open procedure. All consecutive patients who underwent elective RPD or OPD between 1 January 2014 and 31 December 2021, in all 18 Dutch centres for pancreatic surgery, were included (*Fig. 1*). The present study included all RPDs performed in the Netherlands, including the initial RPD procedure at each centre. Of the eight centres that performed RPD, seven participated in the LAELAPS-3 training programme^8^. No RPD procedures were performed in the Netherlands before the study interval. The STROBE guidelines^15^ were used for study design and reporting. The study protocol was approved by the scientific committee of the Dutch Pancreatic Cancer Group^16^. Ethical approval was waived by the institutional review board at the Amsterdam UMC due to coded data use.

### Eligibility

Included were adult patients who underwent elective RPD or OPD for any pancreatic or peri-ampullary disease. Patients who underwent hybrid procedures (for example robotic resection with pancreatojejunostomy or hepatojejunostomy performed via laparotomy) were excluded, as were patients with chronic pancreatitis or cholangitis as an indication for surgery, planned/intended arterial resection, insufficient baseline data, or missing primary outcome data.

### Primary and secondary outcomes

The primary outcomes were major complications (Clavien–Dindo grade greater than or equal to III) and in-hospital/30-day mortality^17^. Secondary outcomes included intraoperative parameters (for example operating time and intraoperative blood loss), procedure-specific complications (for example postoperative pancreatic fistula (POPF) and re-interventions), duration of hospital stay, and oncological outcomes (for example R0 resection rate and number of lymph nodes resected).

### Surgical technique and definitions

In the Netherlands, RPD was implemented through a nationwide training programme using a standardized technique, based on the Pittsburgh approach^8^. The anastomosis technique in OPD was not standardized and based on local preference. All of the included centres, except for one, placed surgical drains after RPD and OPD. Preoperative variables included baseline characteristics, co-morbidities, preoperative imaging information for vascular/organ involvement (CT/MRI), ASA grade^18^, and Eastern Cooperative Oncology Group (ECOG) performance status^19^. Conversion during RPD was recorded if a laparotomy was performed for a reason other than specimen extraction^20^. The International Study Group on Pancreatic Surgery (ISGPS) definitions were used to classify POPF^21^, delayed gastric emptying (DGE)^22^, post-pancreatectomy haemorrhage (PPH)^23^, and chyle leak^24^. The International Study Group of Liver Surgery (ISGLS) grading system was used to define bile leakage^25^. Only clinically relevant complications (that is grade B/C) were included. The diagnosis of wound infection, pneumonia, and organ failure was based on clinical features; no predefined diagnosis was adapted in the DPCA. Failure to rescue was defined as the death of a patient due to a major postoperative complication^26,27^. Resection margin status was classified as microscopic radical resection (greater than 1 mm; R0), microscopic irradical (less than or equal to 1 mm; R1), or macroscopic margin involvement (R2). The DPCA collects outcomes during the entire hospital stay (that is regardless of duration) and up to 30 days after surgery in case of earlier discharge. For each patient, the baseline risk of POPF grade B/C was determined using the updated adjusted Fistula Risk Score (ua-FRS), which is validated for both open and minimally invasive PD^28^. The calculated scores were then assigned to one of three risk groups: low risk (less than or equal to 5%); moderate risk (6%–20%); and high risk (greater than 20%)^28^.

### Propensity-score matching

Propensity-score matching was used to minimize treatment allocation bias^29^. The two treatment groups (RPD and OPD) were matched in a 1 : 1 ratio (standard caliper width of 0.1) on a set of predefined variables that may confound the comparisons. Covariates associated with the probability of undergoing RPD for each patient (that is the propensity score) were obtained from a logistic regression model (*P* < 0.100) and known cofounders were added (Appendix Table S1). The final covariates were age, BMI, ASA grade, sex, preoperative tumour size, vascular involvement on preoperative imaging, suspected malignancy, neoadjuvant therapy, year of surgery, preoperative fistula risk parameters (pancreatic texture and duct diameter on preoperative imaging), volume, and whether the PORSCH algorithm was implemented in the treatment centre. The PORSCH trial was a nationwide trial investigating an algorithm for the early detection and minimally invasive step-up management of patients after pancreatic resection, which reduced postoperative mortality^30^; this postoperative algorithm is currently still used in all centres included in the present study.

### Sensitivity and subgroup analyses

A total of four sensitivity analyses were conducted. First, the impact of the learning curve on outcomes was assessed by excluding the first learning curve phase by excluding the first 30 RPDs per centre. Second and third, the impact of high-volume and lower-volume centres on outcomes was assessed by excluding RPDs from centres performing less than 20 RPDs annually (the recommended minimum annual volume per the Miami guidelines^13^) and greater than or equal to 20 RPDs annually respectively. Years were calculated starting from the date of the first RPD procedure at each centre. Fourth, the impact of pancreatic ductal adenocarcinoma (PDAC) on outcomes was assessed by excluding all indications other than PDAC. The association between the RPD sensitivity analyses and primary outcomes was assessed using ORs for major complications and in-hospital/30-day mortality. Last, the effect of major complications on postoperative recovery was assessed by determining the duration of hospital stay after RPD and OPD for patients with and without major complications.

### Statistical analysis

Data were analysed using SPSS^®^ (IBM, Armonk, NY, USA; version 28.0) or the R programming environment (Rstudio), with propensity-score matching performed using the Rstudio Matching package (caliper 0.1).

All patients were analysed according to the intention-to-treat principle, hence conversions from RPD to OPD were included in the RPD group. The initially intended approach (RPD or OPD) is recorded in the DPCA. Continuous data are expressed as mean(s.d.) or median (interquartile range (i.q.r.)) and were compared using the two independent sample *t* test or the Mann–Whitney *U* test, as appropriate. Categorical data are presented *n* (%) and were compared using the chi-squared test or Fisher’s exact test, as appropriate. Additionally, log rank tests on Kaplan–Meier estimates were used to compare hospital stay between the groups of patients with and without major complications.

The standardized mean difference (SMD) was used to assess balance at baseline between groups, before and after propensity-score matching; small absolute values (less than 0.1) indicate balance. Missing baseline data of variables used for propensity-score matching were resolved by imputing five sets using multiple imputation with predictive mean matching (Appendix Table S2). Outcome data were not imputed. Subsequently, propensity-score matching was applied to the multiple imputed data sets in a 1 : 1 ratio without replacement. Descriptive statistics were generated by averaging the values across the imputed data sets according to Rubin’s rules and *P* values were computed by applying logistic regression models to the imputed data sets and subsequently pooling the causal effect estimates^31^. Statistical significance was set at *P* ≤ 0.050; all tests were two-sided.

## Results

Overall, 733 patients who underwent RPD and 4641 who underwent OPD were included from 18 Dutch Pancreatic Surgery Group centres (*Fig. 1*); 8 centres started performing RPD during the study interval. The nationwide use of RPD among all PDs increased from 2.5% (14) in 2016, when the first centre started implementing RPD, to 24.9% (200) in 2021, when eight centres were performing RPD. After exclusion, 701 RPD patients from 8 centres and 4447 OPD patients from 18 centres were included. The median annual total PD volume (RPD and OPD combined) was 44 (i.q.r. 33–80) among the eight centres performing RPD and 26 (i.q.r. 23–34) among the centres that only performed OPD. The median annual volume of RPD was 20 (i.q.r. 16–27), which included the first RPD performed at every centre. In the final two study years (2020–2021), five of eight RPD centres met the Miami volume cut-off of 20 RPDs per year, whereas the other centres performed between 7 and 19 RPDs annually. The median total RPD experience was 86 procedures per centre (range 48–149). The same surgical team performed the RPD procedures in every centre. Of the eight centres, three had experience with laparoscopic PD before starting RPD. Of all included patients, 698 of 701 patients who underwent RPD were matched (1 : 1) to an OPD control.

### Baseline characteristics

*Table 1* shows the baseline characteristics before and after matching. Before matching, in the RPD group, less vascular involvement was seen on preoperative imaging (15% *versus* 28%; SMD −0.32) and fewer patients received neoadjuvant chemotherapy (8.6% *versus* 10.5%; SMD 0.11). The median ua-FRS was higher in the RPD group than in the OPD group (34 (i.q.r. 20–49) *versus* 25 (i.q.r. 14–42) respectively; SMD 0.25). More RPD procedures than OPD procedures were performed during or after the PORSCH trial (70% *versus* 42%; SMD 0.58). After propensity-score matching, most differences in baseline variables were minimized.

**Table 1:** Baseline characteristics of patients who underwent robotic pancreatoduodenectomy or open pancreatoduodenectomy, before and after propensity-score matching

	Full cohort, before propensity-score matching				Study cohort, after propensity-score matching			
	RPD (*n* = 701)	OPD (*n* = 4447)	SMD	Variance ratio	RPD (*n* = 698)	OPD (*n* = 698)	SMD	Variance ratio
**Patient characteristics**								
Age (years), median (i.q.r.)	69 (62–75)	69 (61–74)	0.09	0.97	69 (62–75)	69 (61–75)	0.01	1.08
Mean(s.d.)	67.9(10.1)	67.0(10.3)			67.8(10.6)	67.9(10.0)		
Female	316 (45.1)	2000 (45.0)	−0.01		315 (45.1)	319 (45.7)	−0.02	
BMI (kg/m^2^), median (i.q.r.)	25.1 (22.7–27.8)	24.7 (22.3–27.5)	0.08	0.84	25.1 (22.7–27.7)	25.2 (22.7–27.9)	0.01	0.81
BMI >30 kg/m^2^	91 (13.0)	552 (12.8)			91 (13.0)	105 (15.0)		
BMI >35 kg/m^2^	19 (2.7)	135 (3.0)			28 (3.6)	19 (2.7)		
ASA grade								
I	44 (6.3)	437 (9.8)	−0.12		45 (6.4)	46 (6.6)	−0.03	
II	406 (57.9)	2628 (59.1)			422 (60.4)	413 (59.2)		
III	219 (31.2)	1200 (27.0)			228 (32.7)	232 (33.2)		
IV	2 (0.3)	32 (0.7)			3 (0.4)	9 (1.3)		
Unknown	30 (4.3)	150 (.3.4)			–	–		
ECOG performance status								
0–1	524 (74.8)	3374 (75.9)	−0.13		521 (74.6)	483 (69.2)	−0.06	
2	19 (2.7)	294 (6.6)			19 (2.7)	40 (5.7)		
3	3 (0.4)	50 (1.1)			3 (0.4)	5 (0.7)		
4	-	2 (0.01)			-	-		
Unknown	155 (21.8)	727 (16.3)			154 (20.5)	169 (24.2)		
Updated adjusted Fistula Risk Score, median (i.q.r.)	34 (20–49)	25 (14–42)	0.25	1.00	33 (18–48)	30 (17–47)	0.02	1.00
Fistula risk categories								
Low risk ≤5%	5 (0.7)	77 (1.7)	−0.32		5 (0.6)	5 (0.7)	−0.01	
Moderate risk 6–20%	145 (20.7)	1098 (24.7)			196 (28.1)	198 (28.4)		
High risk >20%	416 (59.3)	1967 (44.2)			497 (71.2)	495 (70.9)		
Unknown due to missing variables	135 (19.3)	1305 (29.3)			–	–		
Included in/after PORSCH trial	487 (69.5)	1851 (41.6)	0.58		484 (69.3)	490 (70.2)	−0.02	
**Preoperative tumour characteristics**								
Localization								
Pancreas	332 (47.3)	2270 (51.0)	0.10		330 (47.3)	323 (46.3)	0.10	
Peri-ampullary or CBD	121 (17.3)	531 (11.9)			120 (17.1)	138 (19.7)		
Duodenum	40 (5.7)	256 (5.8)			40 (5.7)	52 (7.5)		
Unknown	208 (29.7)	1390 (31.3)			208 (29.8)	132 (29.8)		
Suspected malignancy	511 (72.9)	3769 (84.8)	0.03		552 (79.1)	570 (81.7)	0.02	
Preoperative tumour size (mm), median (i.q.r.)	25 (18–35)	26 (22–35)	0.04	1.13	25 (18–35)	25 (19–34)	0.02	1.80
Vascular involvement on preoperative imaging								
No	571 (81.5)	3043 (68.4)			588 (84.2)	589 (84.4)		
Yes	106 (15.1)	1238 (27.8)	−0.32		110 (15.8)	108 (15.5)	0.07	
Unknown	24 (3.4)	166 (3.7)			–	–		
Neoadjuvant therapy received	60 (8.6)	469 (10.5)	0.11		81 (11.6)	91 (13.0)	0.04	
**Pathology**								
Histological diagnosis								
Adenocarcinoma*	481 (68.6)	3505 (78.9)	−0.11		474 (67.9)	512 (73.4)	−0.02	
Pancreas	218 (31.1)	1988 (44.7)	−0.17		218 (31.2)	246 (35.2)	−0.15	
Distal bile duct	97 (13.8)	562 (12.6)	0.09		96 (13.8)	98 (14.0)	−0.09	
Ampulla	118 (16.8)	571 (12.8)	0.14		118 (16.9)	107 (15.3)	0.10	
Duodenum/other	40 (5.7)	346 (7.8)	−0.06		42 (6.0)	61 (8.7)	−0.10	
NET	49 (7.0)	196 (4.4)	0.09		48 (6.9)	35 (5.0)	0.10	
IPMN	89 (12.7)	307 (6.9)	0.17		88 (12.6)	58 (8.3)	0.12	
Intestinal adenoma	39 (5.6)	108 (2.4)	0.14		39 (5.6)	33 (4.7)	0.07	
Other/unknown	41 (6.0)	331 (7.4)	−0.10		41 (5.9)	52 (7.5)	−0.15	
Tumour size (mm), median (i.q.r.)	25 (16–34)	28 (20–38)	−0.20		25 (16–35)	26 (18–38)	−0.17	

Values are *n* (%) unless otherwise indicated. *Pancreatic ductal, duodenum, distal bile duct, or other type. RPD, robotic pancreatoduodenectomy; OPD, open pancreatoduodenectomy; SMD, standardized mean difference; i.q.r., interquartile range; ECOG, Eastern Cooperative Oncology Group; CBD, common bile duct; NET, neuroendocrine tumour; IPMN, intraductal papillary mucinous neoplasm.

### Operative outcomes

After matching, some differences in operative outcomes were observed (*Table 2*), with more often a pylorus-resecting procedure (79.2% *versus* 52.0%; *P* < 0.001), a longer operating time (median of 359 *versus* 301; *P* < 0.001), a lower estimated blood loss (median of 200 *versus* 500; *P* < 0.001), and fewer venous resections (4.6% *versus* 8.5%; *P* = 0.007) for RPD compared with OPD. The overall conversion rate was 7.3%.

**Table 2:** Operative findings and outcomes of patients who underwent robotic pancreatoduodenectomy or open pancreatoduodenectomy, before and after matching

	Full cohort, before propensity-score matching			Study cohort, after propensity-score matching		
	RPD (*n* = 701)	OPD (*n* = 4447)	*P*	RPD (*n* = 698)	OPD (*n* = 698)	*P*
**Type of resection**						
Pylorus-preserving PD	145 (20.7)	2435 (54.8)	<0.001*	145 (20.8)	335 (48.0)	<0.001*
Pylorus-resecting PD	556 (79.3)	2012 (45.3)		553 (79.2)	362 (52.0)	
Operating time (min), median (i.q.r.)	359 (304–424)	312 (249–388)	<0.001*	359 (303–424)	301 (243–375)	<0.001*
Estimated blood loss (ml), median (i.q.r.)	200 (100–450)	500 (300–1000)	<0.001*	200 (100–450)	500 (265–900)	<0.001*
Conversion	51 (7.3)	NA		51 (7.3)	NA	
**Pancreas texture**						
Hard/firm	170 (24.3)	1544 (34.7)	<0.001*	195 (27.9)	207 (29.7)	0.556
Normal/soft	462 (65.9)	2439 (54.8)		502 (71.9)	490 (70.2)	
Unknown	69 (9.7)	464 (11.5)		–	–	
**Venous resection†**						
Wedge	32 (4.5)	465 (10.5)	<0.001*	32 (4.6)	59 (8.5)	0.007*
Segment	16 (2.2)	277 (6.2)	<0.001*	16 (2.3)	24 (3.4)	0.327
Intraoperative drain placement	640 (91.8)	4293 (97.8)	<0.001*	638 (91.4)	676 (96.8)	<0.001*
Octreotide/pasireotide	458 (65.7)	2642 (60.4)	0.008*	456 (65.3)	416 (59.6)	0.010*
**Oncological outcomes‡**	*n* = 218/701	*n* = 1988/4447		*n* = 213/698	*n* = 240/698	
R margin						
R0	128 (61.5)	1024 (53.8)	0.033*	128 (60.1)	131 (54.6)	0.106
R1/R2 resection	80 (36.7)	880 (44.3)	0.092	80 (36.9)	108 (45.0)	0.234
Unknown/missing	10 (4.6)	84 (4.2)		5 (4.6)	7 (2.9)	
Lymph nodes						
Total resected, mean(s.d.)	15 (6)	16 (8)	0.044*	14 (5)	15 (7)	0.008*
Ratio, median (i.q.r.)	0.05 (0–0.2)	0.09 (0–0.3)	0.001*	0.05 (0–0.2)	0.08 (0–0.2)	0.386

Values are *n* (%) unless otherwise indicated. *P* values are for the differences between RPD and OPD before and after propensity-score matching. *Statistically significant. †Such as porto-mesenteric vein and superior mesenteric vein. ‡Pancreatic ductal adenocarcinoma only. RPD, robotic pancreatoduodenectomy; OPD, open pancreatoduodenectomy; PD, pancreatoduodenectomy; i.q.r., interquartile range; NA, not applicable.

### Primary outcomes

*Table 3* shows the details of the primary outcomes before and after matching. After matching, no significant differences in major complications (40.3% *versus* 36.2%; *P* = 0.186) and in-hospital/30-day mortality (4.1% *versus* 3.0%; *P* = 0.326) were found between RPD and OPD respectively.

**Table 3:** Postoperative outcomes (30-day) of patients who underwent robotic pancreatoduodenectomy or open pancreatoduodenectomy, before and after matching

	Full cohort, before propensity-score matching			Study cohort, after propensity-score matching		
	RPD (*n* = 701)	OPD (*n* = 4447)	*P*	RPD (*n* = 698)	OPD (*n* = 698)	*P*
**Morbidity**						
Major complications (CD grade ≥III)	283 (40.4)	1324 (29.8)	<0.001*	281 (40.3)	253 (36.2)	0.186
CD grade IIIa	174 (24.8)	833 (18.7)	<0.001*	172 (25.1)	170 (24.8)	0.811
CD grade IIIb	59 (8.4)	257 (5.8)	0.004*	59 (8.7)	43 (6.3)	0.171
CD grade IV	27 (3.9)	166 (3.7)	0.878	27 (3.7)	27 (3.7)	0.991
In-hospital/30-day mortality	28 (4.0)	148 (3.3)	0.363	28 (4.0)	21 (3.1)	0.326
Failure to rescue, %	9.2	10.0	0.398	9.2	7.9	0.484
**Re-interventions**						
Radiological	227 (32.4)	894 (20.1)	<0.001*	225 (32.2)	196 (28.0)	0.203
Endoscopic	75 (10.7)	286 (6.4)	<0.001*	74 (10.6)	59 (8.5)	0.356
Surgical reoperation	68 (9.7)	336 (7.6)	0.061	67 (9.2)	51 (7.3)	0.170
**POPF grade B/C**	176 (25.1)	694 (15.6)	<0.001*	174 (24.9)	164 (23.5)	0.578
Grade C	13 (1.9)	111 (2.5)	0.304	13 (1.9)	16 (2.3)	0.617
PPH grade B/C	88 (12.6)	334 (7.5)	<0.001*	87 (12.5)	67 (9.6)	0.111
DGE grade B/C	155 (22.1)	860 (19.3)	0.081	154 (22.1)	156 (22.3)	0.959
Bile leak grade B/C	59 (8.4)	219 (4.9)	<0.001*	59 (8.5)	42 (6.0)	0.135
Chyle leak	20 (2.9)	217 (6.4)	<0.001*	19 (2.7)	47 (6.7)	0.007*
Pneumonia	43 (6.1)	170 (4.7)	0.089	43 (6.1)	37 (5.3)	0.732
Wound infection	52 (7.4)	400 (9.0)	0.008*	52 (7.4)	85 (12.2)	0.008*
Transfusion during admission	149 (21.3)	754 (17.0)	0.003*	148 (21.2)	139 (19.9)	0.513
Duration of hospital stay (days), median (i.q.r.)	11 (7–19)	12 (8–19)	<0.001*	11 (7–19)	12 (8–19)	<0.001*
Readmission	147 (21.0)	777 (16.3)	0.017*	147 (21.1)	144 (20.6)	0.296

Values are *n* (%) unless otherwise indicated. *P* values are for the differences between RPD and OPD before and after propensity-score matching. *Statistically significant. RPD, robotic pancreatoduodenectomy; OPD, open pancreatoduodenectomy; CD, Clavien–Dindo; POPF, postoperative pancreatic fistula; PPH, post-pancreatectomy haemorrhage; DGE, delayed gastric emptying; i.q.r., interquartile range.

### Postoperative outcomes

After matching, no differences were observed in the rates of POPF grade B/C (24.9% *versus* 23.5%; *P* = 0.578), PPH grade B/C (12.5% *versus* 9.6%; *P* = 0.111), and DGE grade B/C (22.1% *versus* 22.3%; *P* = 0.959) after RPD and OPD respectively (*Table 3*). Lower rates of chyle leak grade B/C (2.7% *versus* 6.7%; *P* = 0.007) and wound infections (7.4% *versus* 12.2%; *P* = 0.008) were found after RPD. The rates of radiological intervention (32.2% *versus* 28.0%; *P* = 0.203) and surgical reoperation (9.2% *versus* 7.3%; *P* = 0.170) did not differ significantly between the RPD group and the OPD group respectively. Overall, the median hospital stay was shorter after RPD (11 days) compared with after OPD (12 days) (*P* < 0.001).

### Sensitivity and subgroup analyses

*Table 4* shows the study outcomes of the primary and sensitivity analyses of the RPD cohort and *Appendix Fig. S1* shows the impact of the sensitivity analyses on primary outcomes after RPD and OPD.

**Table 4:** Sensitivity analyses for the robotic pancreatoduodenectomy cohort

	Full RPD cohort (*n* = 701)	Excluding the first 30 RPD cases per centre (*n* = 466)	High-volume centres (≥20 RPDs/year) (*n* = 523)	Lower-volume centres (<20 RPDs/year) (*n* = 178)	PDAC only (*n* = 218)
**Intraoperative outcomes**					
Operating time (min), median (i.q.r.)	367 (314–429)	357 (297–420)	369 (327–4341)	324 (306–427)	351 (284–4467)
Estimated blood loss (ml), median (i.q.r.)	211 (100–500)	200 (100–400)	200 (100–400)	250 (100–550)	300 (163–500)
Conversion	51 (7.3)	26 (5.6)	33 (6.3)	20 (11.2)	17 (7.8)
**Postoperative outcomes (30-day)**					
Major complication (CD grade ≥III)	284 (40.5)	198 (42.5)	227 (43.4)	60 (33.7)	63 (28.9)
CD grade IIIa	174 (24.8)	118 (25.3)	146 (27.9)	28 (15.7)	38 (17.4)
CD grade IIIb	59 (8.4)	43 (9.2)	46 (8.8)	13 (7.3)	13 (6.0)
CD grade IV	27 (3.9)	18 (4.1)	20 (3.8)	6 (3.4)	4 (1.8)
In-hospital/30-day mortality	28 (4.0)	19 (4.1)	15 (2.9)	13 (7.3)	8 (3.7)
Failure to rescue, %	9.2	9.6	6.6	21.6	12.7
Re-interventions					
Radiological	227 (32.4)	160 (34.3)	189 (36.1)	38 (21.3)	43 (19.7)
Endoscopic	75 (10.7)	52 (11.2)	59 (11.3)	16 (9.0)	18 (19.7)
Surgical reoperation	68 (9.7)	43 (9.2)	47 (9.0)	21 (11.8)	13 (6.0)
POPF grade B/C	176 (25.1)	133 (28.5)	148 (28.3)	28 (15.7)	19 (8.7)
Grade C	13 (1.9)	6 (1.3)	6 (1.1)	7 (3.9)	2 (0.9)
PPH grade B/C	88 (12.6)	64 (13.7)	61 (11.7)	27 (15.2)	24 (11.0)
DGE grade B/C	155 (22.1)	97 (20.8)	110 (21.0)	45 (25.3)	34 (15.6)
Bile leak grade B/C	59 (8.4)	40 (6.8)	50 (9.6)	92 (5.1)	11 (5.0)
Chyle leak	20 (2.9)	17 (3.6)	18 (3.4)	2 (1.1)	8 (3.7)
Pneumonia	43 (6.1)	132 (6.9)	30 (5.7)	13 (7.3)	15 (6.9)
Surgical-site infections	52 (7.4)	330 (7.1)	34 (6.5)	18 (10.1)	13 (6.0)
Transfusion during admission	149 (21.3)	103 (22.1)	109 (20.8)	40 (22.5)	42 (19.3)
Duration of hospital stay (days), median (i.q.r.)	11 (7–18)	10 (7–21)	11 (7–20)	11 (7–17)	8 (6–15)

Values are *n* (%) unless otherwise indicated. RPD, robotic pancreatoduodenectomy; PDAC, pancreatic ductal adenocarcinoma; i.q.r., interquartile range; CD, Clavien–Dindo; POPF, postoperative pancreatic fistula; PPH, post-pancreatectomy hemorrhage; DGE, delayed gastric emptying.

The first sensitivity analysis regarding the impact of the learning curve (excluding the first 30 RPDs for every centre; 466) showed that it did not influence the rates of major complications, POPF, and mortality. The second sensitivity analysis regarding the impact of high-volume centres (excluding RPDs from centres performing less than 20 RPDs/year; 523) showed that they did influence the rates of major complications, POPF, and mortality. For the third sensitivity analysis, regarding the impact of lower-volume centres, a lower in-hospital/30-day mortality rate (2.9% *versus* 7.3%; *P* = 0.009) and a lower conversion rate (6.3% *versus* 11.2%; *P* = 0.032) were found comparing RPDs from high-volume centres (523) with RPDs from lower-volume centres (178) respectively. The fourth sensitivity analysis, including only patients with PDAC (218), showed that patients with PDAC had lower rates of major complications (28.9% *versus* 40.5%; *P* = 0.002) and POPF (8.7% *versus* 25.1%; *P* < 0.001) compared with the total RPD cohort, with a 7.8% conversion rate and a 3.7% in-hospital/30-day mortality rate.

For patients without major complications (413 RPD and 3059 OPD), the median hospital stay was 8 (i.q.r. 6–12) days after RPD compared with 10 ( i.q.r. 8–14) days after OPD (*P* < 0.001) (*Appendix Fig. S2*). For patients with major complications (283 RPD and 1324 OPD), the median hospital stay was 19 (i.q.r. 13–34) days after RPD compared with 20 (i.q.r. 14–33) days after OPD (*P* = 0.597).

## Discussion

This nationwide propensity-score-matched cohort study provides a comprehensive assessment of the early nationwide experience with RPD compared with conventional OPD in the Netherlands. During the first 6 years of RPD implementation, no differences in major morbidity and in-hospital/30-day mortality were found. RPD was associated with a longer operating time, less intraoperative blood loss, lower rates of wound infection and chyle leak, and a 1 day shorter hospital stay (2 days for patients without major morbidity) compared with OPD. For patients with PDAC, RPD was associated with a similar R0 resection rate, but fewer retrieved lymph nodes. RPD was not associated with an increased risk of POPF; also not when stratified by ua-FRS risk categories. The present study also showed a lower in-hospital/30-day mortality rate (2.9% *versus* 7.3%; *P* = 0.009) and a lower conversion rate (6.3% *versus* 11.2%; *P* = 0.032) in centres performing greater than or equal to 20 RPDs annually compared with centres performing less than 20 RPDs annually.

Studies comparing nationwide outcomes of RPD and OPD from the start of RPD implementation have not yet been reported, making it difficult to contrast the results of the present study with corresponding benchmarks. Zureikat *et al*.^32^ compared 211 patients who underwent RPD in two specialized RPD centres that had completed the learning curve with 817 patients who underwent OPD in six high-volume centres and found no differences in mortality and short-term oncological outcomes. RPD was independently associated with a reduction in major complications, corrected for POPF risk factors, which was not observed in the cohort of the present study. The largest propensity-score-matched multicentre comparison of minimally invasive PD and OPD to date, conducted by the European Consortium on Minimally Invasive Pancreatic Surgery (E-MIPS), found no differences in postoperative mortality, major complications, and hospital stay^33^. However, a higher POPF rate after minimally invasive PD was found, which was no longer present after excluding patients who underwent a single-layer pancreatojejunostomy. Unfortunately, the study by Klompmaker *et al*.^33^ only included 184 RPD procedures. Furthermore, a meta-analysis including 2175 RPD procedures and 10 404 OPD procedures from 24 studies reported superior RPD outcomes regarding blood loss, wound infections, duration of hospital stay, R0 resections, and lymph node retrieval^34^. However, the meta-analysis also included non-matched studies, increasing the risk of selection bias.

The use of RPD is relatively high in the Netherlands (25% in 2021) compared with other national databases (for example 3% in the National Surgical Quality Improvement Program (NSQIP) data set)^35,36^. This rapid implementation of RPD was clearly facilitated by the nationwide LAELAPS-3 training programme. However, the learning curve has not yet been completed in all centres, as the current median total institutional RPD experience ranges from 48 to 149 RPD procedures. Several studies have reported a learning curve of 20–100 RPD procedures^4,37–40^. Therefore, the present study serves more to assess the overall safety of the nationwide implementation of RPD in selected patients and cannot be used to demonstrate or dismiss the superiority of RPD over OPD. To do so, a randomized trial is needed in centres that have completed the learning curve, such as the recently completed single-centre EUROPA pilot trial and the multicentre DIPLOMA-2 trial (ISRCTN27483786) and the ongoing PORTAL trial^41^.

The findings of the present study underscore the complexity of reproducing outcomes achieved by highly specialized pancreatic surgery centres, which benefit from a concentrated caseload and stringent patient selection, on a nationwide level. In contrast, RPD implementation across a country introduces additional variables, including case volume, patient diversity, and perioperative care. Furthermore, it should be noted that the present study included all RPD procedures performed in the Netherlands, including the very first procedure for each centre, most within the LAELAPS-3 training programme. A sensitivity analysis that excluded the first 30 RPDs per centre confirmed the absence of a strong learning curve effect on the results. The value of such a training programme is therefore confirmed.

Some studies reported concerns regarding an increased rate of POPF after minimally invasive PD^33^. Considering the outcomes of the total cohort in the present study, an increased rate of POPF after RPD was found. However, after adjusting for POPF risk factors, RPD was not associated with an increased rate of POPF. Only in the moderate-risk ua-FRS patient group was there a higher rate of POPF after RPD (*Appendix Fig. S3*). Similar results were reported in a similar single-centre propensity-score-matched study on RPD *versus* OPD^42^. Nevertheless, the overall POPF rate in the present study (24%) seems high compared with the 7–28% reported by others^10,42,43^. The high incidence of POPF in both groups in the present study may be partly attributable to the PORSCH trial, resulting in the early detection and minimally invasive treatment of POPF using radiological catheter drainage. The PORSCH algorithm reduced 90-day mortality in the intervention group compared with the control group (3% *versus* 5%; *P* = 0.029) and increased the detection of POPF, with a (non-significant) increase in the use of catheter drainage (29% *versus* 23%; *P* = 0.160)^30^. The PORSCH algorithm may be a contributing factor to the high POPF rate in the cohort of the present study. Additionally, the POPF rate in the matched OPD population was significantly higher than that in the pre-matched OPD cohort, illustrating the high-risk patient selection for RPD. Finally, the four published randomized trials on laparoscopic PD *versus* OPD found no differences in the rate of POPF, although no risk categories for POPF were reported^44–48^.

Finally, although not statistically significant, but of potential clinical relevance, the present study showed a higher margin-negative (R0) resection rate after RPD, both in the overall RPD cohort and in the subgroup analysis for PDAC alone. Conversely, the number of retrieved lymph nodes was significantly lower after RPD (14 *versus* 15 nodes). These contradictory findings could be explained by the residual confounding by indication. The oncological safety of RPD compared with OPD should be studied in future randomized trials, focusing on radical resection rates and, ideally, survival rates.

The present study should be interpreted considering the following limitations. First, although the data were retrieved from the nationwide and mandatory DPCA registry, missing data and therefore information bias could not be avoided. Second, data collection was limited to the established variables, thus limiting the analyses. For example, the following were lacking: reasons for conversion; reasons for reoperations; and mortality beyond 30 days. Third, the retrospective nature of the present study is a limitation, with inherent biases, such as treatment allocation bias. Despite an attempt to minimize the influence of treatment allocation bias, by applying propensity-score matching, outcomes may still have been influenced by unknown confounders. Only a randomized trial can eliminate this bias, such as the recently completed single-centre EUROPA trial (DRKS00020407) and the international multicentre DIPLOMA-2 trial (ISRCTN27483786) and the currently ongoing PORTAL trial^41^. Fourth, data on quality of life, use of adjuvant chemotherapy, and overall survival were not available for the present study. Clearly, these data are highly relevant and randomized trials will provide these outcomes. The strengths of the present study include its nationwide multicentre design, the inclusion of the very first RPD patient for each centre, the large study size, and the propensity-score matching, aiming to minimize selection bias.

## Supplementary Material

znae043_Supplementary_Data

## Data Availability

The data that support the findings of this study are available on request from the corresponding author. The data are not publicly available due to privacy and ethical restrictions.
