# Supplementary material for: Early experience with robotic pancreatoduodenectomy versus open pancreatoduodenectomy: nationwide propensity-score-matched analysis
Source: Br J Surg. 2024 Feb 27;111(2):znae043. doi: 10.1093/bjs/znae043 (PMC10898866; doi:10.1093/bjs/znae043)
Supplement: znae043_Supplementary_Data [file znae043_supplementary_data.docx]

# **Early experience of robotic versus open pancreatoduodenectomy: a nationwide propensity-score matched analysis**

*Original article*

Nine de Graaf, MD^1,2,3^, Maurice J.W. Zwart, MD^1,2^, Jony van Hilst, MD PhD^1,2,4^, Bram van den Broek, BSc^5^, Bert A. Bonsing, MD PhD^6^, Olivier R. Busch, MD PhD^1,2^*,* Peter-Paul L.O. Coene, MD PhD^7^, Freek Daams, MD PhD^1,2^, Susan van Dieren, PhD ^1,2,8^, Casper H.J. van Eijck, MD PhD^5^, Sebastiaan Festen, MD PhD^4^, Ignace H.J.T. de Hingh, MD PhD^9^, Daan J. Lips, MD PhD^10^, Misha D.P. Luyer, MD PhD^9^, J. Sven D. Mieog, MD PhD^6^, Hjalmar C. van Santvoort, MD PhD^11,12^, George P. van der Schelling, MD PhD^13^, Martijn W.J. Stommel, MD PhD^14^, Roeland F. de Wilde, MD PhD^5^, I. Quintus Molenaar, MD PhD*^11, 12^, Bas Groot Koerkamp, MD PhD*^5^, Marc G. Besselink, MD PhD*^1,2^ for the Dutch Pancreatic Cancer Group

^1^Amsterdam UMC, location University of Amsterdam, Department of surgery, Amsterdam, the Netherlands

^2^Cancer Centre Amsterdam, the Netherlands

^3^Department of General Surgery, Fondazione Poliambulanza Istituto Ospedaliero, Brescia, Italy

^4^Department of Surgery, OLVG, Amsterdam, the Netherlands

^5^Department of Surgery, Erasmus MC Cancer Institute, Rotterdam, the Netherlands

^6^Department of Surgery, Leiden University Medical Centre, Leiden, the Netherlands

^7^Department of Surgery, Maasstad Ziekenhuis, Rotterdam, the Netherlands

^8^Epidemiologist department of Surgery, Amsterdam UMC, location AMC, Amsterdam, The Netherlands ^9^Department of Surgery, Catharina Hospital, Eindhoven, the Netherlands

^10^Department of Surgery, Medisch Spectrum Twente, Enschede, the Netherlands

^11^Department of Surgery, St. Antonius Hospital, the Netherlands

^12^Department of Surgery, Regional Academic Cancer Centre Utrecht, University Medical Centre Utrecht, Utrecht, the Netherlands

^13^Department of Surgery, Amphia Ziekenhuis, Breda, the Netherlands

^14^Deptartment of Surgery, Radboud University Medical Centre, Nijmegen, the Netherlands

**Shared senior authorship*

**Corresponding author:**

Prof. Marc G Besselink

Amsterdam UMC, location University of Amsterdam

Department of Surgery, Cancer Centre Amsterdam,

De Boelelaan 1117 (VUMC hospital, ZH-7F), 1081 HV Amsterdam, the Netherlands

Tel: +31-20-4444400 Email: [m.g.besselink@amsterdamUMC.nl](mailto:m.g.besselink@amsterdamUMC.nl)

**During review:**

Nine de Graaf (study coordinator)

Email: [n.degraaf@amsterdamUMC.nl](mailto:n.degraaf@amsterdamUMC.nl)

**Supplementary Materials - Index**

| **Supplementary Figures and Tables** |  |
| --- | --- |
| Supplementary table 1 | *page 3* |
| Supplementary table 2 | *page 4* |
| Supplementary figure 1 | *page 5* |
| Supplementary figure 2 | *page 6* |
| Supplementary table 3 | *page 7* |
| Supplementary table 4 | *page 7* |
| Supplementary figure 3 | *page 8* |
|  |  |

**Supplementary Figures and Tables**

### Supplementary table 1. Logistic regression model for approach (RPD;OPD) in 5148 unmatched patients undergoing pancreatoduodenectomy

| **Variables obtained from logistic regression model** | B | P-value | Exp (B) | 95% CI lower | 95% CI upper |
| --- | --- | --- | --- | --- | --- |
| Vascular contact | -0,785 | <,001 | 0,456 | 0,367 | 0,567 |
| Preoperative tumor diameter | -0,01 | 0,038 | 0,99 | 0,98 | 0,999 |
| Suspected malignancy | -0,815 | <,001 | 0,442 | 0,296 | 0,661 |
| Year of surgery | 0,581 | <,001 | 1,788 | 1,569 | 2,038 |
| Volume group median <29/y (ref) |  | <,001 |  |  |  |
| Volume group median 30-59/y | -1,772 | <,001 | 0,17 | 0,116 | 0,249 |
| Volume group median >60/y | -0,899 | <,001 | 0,407 | 0,285 | 0,582 |
| PORSCH implementation | -0,887 | <,001 | 0,412 | 0,258 | 0,659 |
| Fistula Risk score | 1,368 | <,001 | 3,927 | 1,784 | 8,642 |
|  |  |  |  |  |  |
|  |  |  |  |  |  |
| **Added confounders** |  |  |  |  |  |
| BMI |  |  |  |  |  |
| Age at surgery |  |  |  |  |  |
| Sexx |  |  |  |  |  |
| ASA score |  |  |  |  |  |
| Neoadjuvant therapy |  |  |  |  |  |
|  |  |  |  |  |  |

*Supplementary table 2. Overview of missing baseline variables in 5148 unmatched patients undergoing pancreatoduodenectomy*

|  |  |  |  |  |  |
| --- | --- | --- | --- | --- | --- |
| \| Surgical approach \| OPD \|  \| RPD \|  \| \| --- \| --- \| --- \| --- \| --- \| \|  \| N \|  \| N \|  \| \|  \| Valid \| Missing \| Valid \| Missing \| \| age at surgery \| 4442 \| 5 \| 701 \| 0 \| \| BMI \| 4324 \| 123 \| 690 \| 11 \| \| sex \| 4446 \| 1 \| 699 \| 2 \| \| asascore \| 4297 \| 150 \| 671 \| 30 \| \| ecogscore \| 4447 \| 0 \| 701 \| 0 \| \| PORSCH implementation \| 4447 \| 0 \| 701 \| 0 \| \| neoadjuvant therapy \| 4447 \| 0 \| 701 \| 0 \| \| preoperative tumor diameter \| 2662 \| 1785 \| 392 \| 309 \| \| vascular contact \| 4281 \| 166 \| 677 \| 24 \| \| suspected malignancy \| 4447 \| 0 \| 701 \| 0 \| \| Volume group median/y \| 4447 \| 0 \| 701 \| 0 \| \| year of surgery \| 4447 \| 0 \| 701 \| 0 \| \| diameter PD \| 3147 \| 1300 \| 574 \| 127 \| \| pancreatic texture \| 3983 \| 464 \| 632 \| 69 \| |  |  |  |  |  |

### Supplementary figure 1. Impact of sensitivity analyses on primary outcomes after RPD and OPD


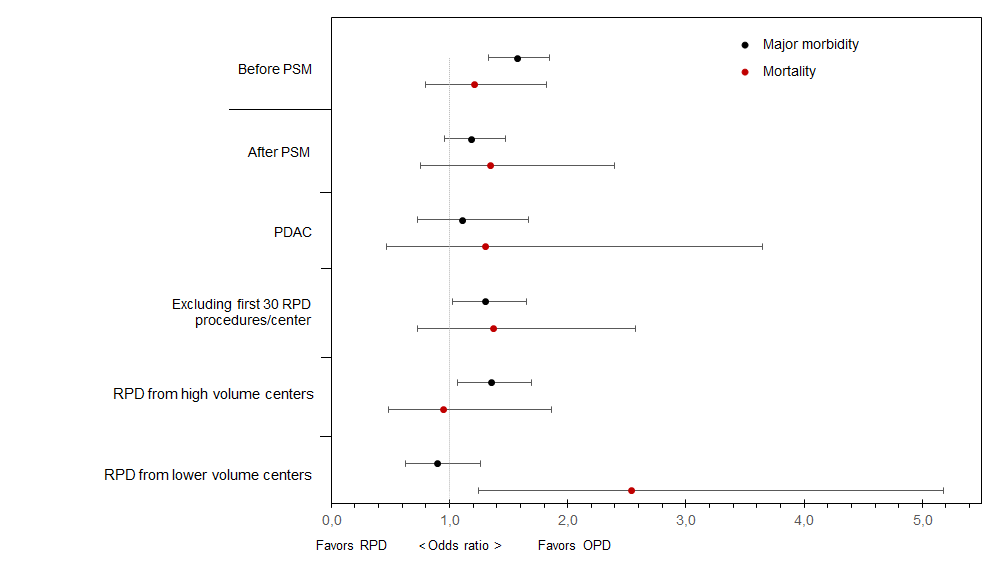


Legend: RPD, robotic pancreatoduodenectomy; OPD, open pancreatoduodenectomy; PSM, propensity-score matching; PDAC, Pancreatic Ductal Adenocarcinoma; high volume, ≥20 RPD/year; lower volume, <20 RPD/year. Dot and error bars indicate point estimate and 95% CI.

*Supplementary figure 2. Hospital stay after RPD and OPD for patients without (A) and with (B) major complications (Clavien-Dindo ≥ 3)*


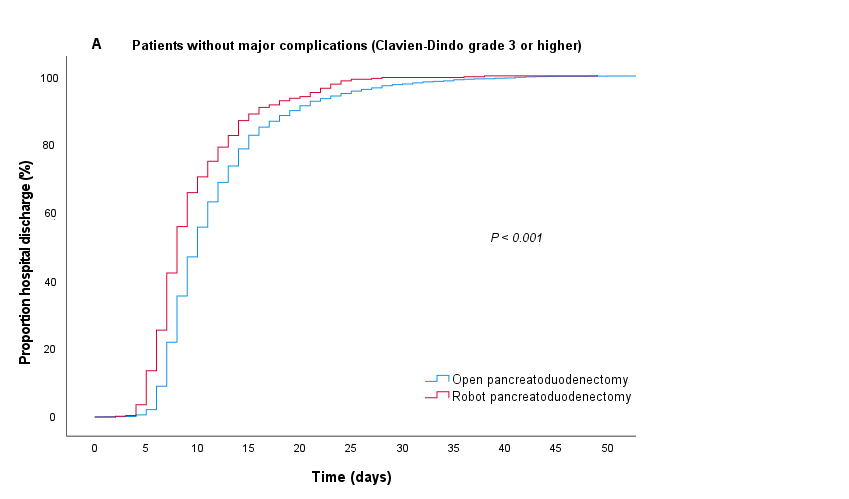

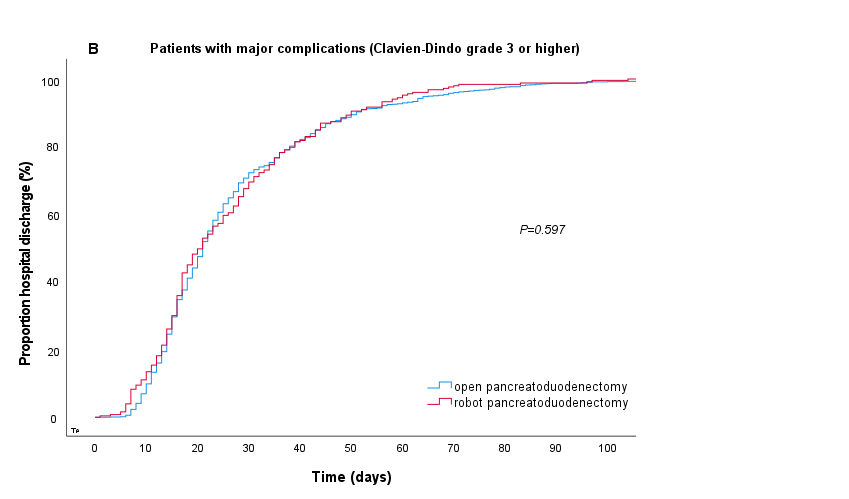


### Supplementary figure 3. Incidence of grade B/C POPF per ua-FRS category in RPD and OPD

*
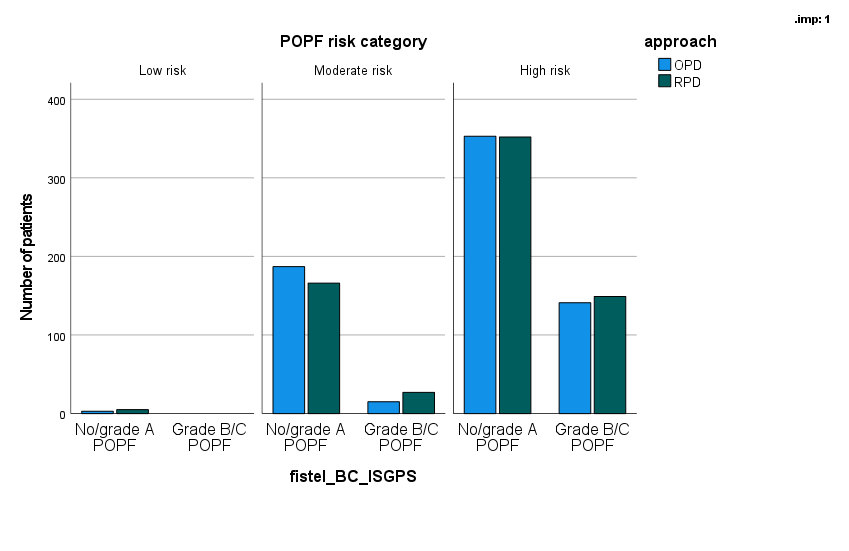
*

Legend: POPF within these groups after RPD vs OPD was 0% vs 0% for low-risk patients, 10.6% vs 4.9% for moderate risk patients (P=.070) and 29.7% vs 30.8% (P=.740) for high-risk patients, respectively
